# Chronic Traffic-Related Air Pollution and Stress Interact to Predict Biologic and Clinical Outcomes in Asthma

**DOI:** 10.1289/ehp.11076

**Published:** 2008-02-27

**Authors:** Edith Chen, Hannah M. C. Schreier, Robert C. Strunk, Michael Brauer

**Affiliations:** 1 Department of Psychology, University of British Columbia, Vancouver, British Columbia, Canada; 2 Department of Pediatrics, Division of Allergy and Pulmonary Medicine, St. Louis Children’s Hospital, Washington University in St. Louis School of Medicine, St. Louis, Missouri, USA; 3 School of Environmental Health, University of British Columbia, Vancouver, British Columbia, Canada

**Keywords:** air pollution, asthma, immune, psychosocial, stress, traffic

## Abstract

**Background:**

Previous research has documented effects of both physical and social environmental exposures on childhood asthma. However, few studies have considered how these two environments might interact to affect asthma.

**Objective:**

This study aimed to test interactions between chronic exposure to traffic-related air pollution and chronic family stress in predicting biologic and clinical outcomes in children with asthma.

**Method:**

Children with asthma (*n* = 73, 9–18 years of age) were interviewed about life stress, and asthma-relevant inflammatory markers [cytokine production, immunoglobulin E (IgE), eosinophil counts] were measured. Parents reported on children’s symptoms. Children completed daily diaries of symptoms and peak expiratory flow rate (PEFR) measures at baseline and 6 months later. Exposure to traffic-related air pollution was assessed using a land use regression model for nitrogen dioxide concentrations.

**Results:**

NO_2_ by stress interactions were found for interleukin-5 (β for interaction term = −0.31, *p* = 0.02), IgE (interaction β = −0.29, *p* = 0.02), and eosinophil counts (interaction β = −0.24, *p* = 0.04). These interactions showed that higher chronic stress was associated with heightened inflammatory profiles as pollution levels decreased. Longitudinally, NO_2_ by stress interactions emerged for daily diary symptoms (interaction β = −0.28, *p* = 0.02), parent-reported symptoms (interaction β = −0.25, *p* = 0.07), and PEFR (interaction β = 0.30, *p* = 0.03). These interactions indicated that higher chronic stress was associated with increases over time in symptoms and decreases over time in PEFR as pollution levels decreased.

**Conclusions:**

The physical and social environments interacted in predicting both biologic and clinical outcomes in children with asthma, suggesting that when pollution exposure is more modest, vulnerability to asthma exacerbations may be heightened in children with higher chronic stress.

Both the physical and the social environments have long been hypothesized to be important contributors to childhood asthma. Although research identifying the physical and social environmental factors that play a role in asthma has made great strides (Crain et al. 2002; Wright 2005), much of this research exists independently of one another. In contrast, few studies have examined how the physical and social environments might interact to affect asthma outcomes. For example, certain combinations of physical and social environmental exposures might produce unique effects beyond exposure to one of these factors alone. Hence, the goal of the present study was to empirically test interactive effects of the physical and social environment to gain a more comprehensive picture of how the environment, broadly defined, contributes to childhood asthma.

Within the literature on physical environment, exposures such as traffic-related air pollution have been repeatedly linked to increased respiratory symptoms, increased asthma-related hospitalizations, and the diagnosis of asthma (Brauer et al. 2007; Edwards et al. 1994; Studnicka et al. 1997). In addition, experimental exposure to markers of traffic-related air pollution such as diesel exhaust particles or nitrogen dioxide increases levels of inflammatory markers relevant to asthma (Barck et al. 2002; DiazSanchez et al. 1997; Pourazar et al. 2004), suggesting that greater inflammation may underlie the effects found in clinical studies.

Within the literature on social environment, factors such as psychological stress have been linked to wheezing, asthma exacerbations, and the diagnosis of asthma in children (Klinnert et al. 2001; Sandberg et al. 2000; Wright et al. 2002). In addition, high levels of stress have been associated with detrimental biologic profiles, such as greater inflammatory responses after antigen challenge or *in vitro* stimulation of immune cells, among children with or at risk for asthma (Chen et al. 2006; Liu et al. 2002; Wright et al. 2004).

Much of this research, although important in its own right, has proceeded largely along separate lines, with little overlap. Few studies have brought these two areas together to examine how physical and social environments might interact to affect asthma outcomes. Although it is possible that social factors alone could affect asthma outcomes, or that physical factors would override any effect of social factors, a number of researchers have argued that social and physical factors might instead combine to modify health outcomes such as asthma (Weiss and Bellinger 2006).

This interaction between the physical and social environments could mean that negative social factors intensify the effect of physical environment exposures (Gee and Payne-Sturges 2004; Morello-Frosch and Shenassa 2006). For example, the combination of certain physical and social environment risk factors might create a “double exposure,” whereby the combination of factors such as high pollution exposure and high stress is most detrimental to asthma. One recent study (Clougherty et al. 2007) empirically tested the notion of physical by social environment interactions in asthma. This study involved a longitudinal investigation of a birth cohort of children in which traffic-related air pollution (indicated by NO_2_) as well as chronic stress (indicated by exposure to violence) were measured. The risk of asthma incidence was elevated as residential NO_2_ exposure increased, but only among children who had high levels of exposure to violence (Clougherty et al. 2007). In the present study, we sought to determine whether a social by physical exposure interaction would predict outcomes in children with existing asthma. More specifically, we tested whether the physical and social environment would interact to predict biologic markers implicated in asthma, and subsequently, whether their interaction would predict changes in clinical outcomes over time in a sample of children with asthma. Although previous research provides some evidence for asthma onset that suggests such interactions might fit a “double jeopardy” hypothesis, so few previous data address this question in children with asthma that our present study aimed to describe the nature of any interaction patterns, rather than testing specific *a priori* hypotheses.

## Materials and Methods

### Subject

Seventy-three children were recruited from Vancouver, British Columbia, Canada, through advertisements in physicians’ offices, local media, and community settings for an observational study of childhood asthma. Advertisements were placed throughout the Greater Vancouver area, and interested families contacted the laboratory for a screening to determine eligibility. Eligible families were then scheduled for a laboratory visit. Visits occurred throughout the year. Participants were required to be physician-diagnosed with asthma (82% with allergic asthma). Children were 9–18 years of age, fluent in English, free of acute respiratory illness at the time of their visit (by parent and child report), and had no chronic illnesses other than asthma. Children gave written assent, and parents provided written consent. The protocol was approved by the University of British Columbia Research Ethics Board.

### Traffic-related air pollution estimates

A land use regression model developed for the study region (Henderson et al. 2007) provided high-resolution spatial estimates of NO_2_ concentrations as an indicator of chronic exposure to traffic-related air pollution. Briefly, 116 passive samplers to collect NO_2_ were deployed for two 14-day periods at 116 sites in the study area. Mean concentrations during these two periods were highly correlated with and closely approximated annual averages from regulatory monitoring network data.

For each of the 116 measurement sites, 55 variables were generated in a geographic information system, and a linear regression model for NO_2_ was built with the most predictive covariates. For NO_2_, the model (*R* ^2^ = 0.56) included the number of major roads within 100- and 1,000-m radius circular buffers of the measurement sites, the number of secondary roads within a 100-m buffer, the population density within a 2,500-m radius, the amount of commercial land use within 750 m, and elevation. Comparison of model predictions to measured annual average concentrations at 16 government regulatory air-monitoring stations had an *R*^2^ of 0.69. The predictive error for the model was estimated with leave-one-out cross-validation, where each model is repeatedly parametrized on *n* – 1 data points and then used to predict the excluded measurement. The mean difference between predicted and measured values, an estimate of the model error, was 0.0 ppb with a standard deviation of 2.75 ppb (~ 15% of the sample mean).

The resulting surface was smoothed (ArcGis Spatial Analyst, Focal Statistics, Redlands, CA) to remove abrupt changes and edge effects to more accurately reflect the measured effect of proximity to roadways (Gilbert et al. 2003). Using the model, we generated a smooth spatial surface of predicted (annual average) concentrations for the entire study area at a resolution of 10 m. To adjust for temporal variability, we fit the corresponding ambient monitoring network data with monthly dummy variables and a covariate for linear trend using the Times Series Forecasting System from SAS (version 9.1; SAS Institute Inc., Cary, NC, USA). We then applied these month–year adjustment factors to the surface to estimate monthly average concentrations. Using these averages, we then computed individual subject average exposures for the full period during which pollution estimates were taken (1998–2003) as an indicator of chronic traffic-related air pollution. We included the full period because the land use regression model is best suited for long-term exposures (Marshall et al. 2008; Nethery et al. 2007), as it is based on spatial differences in land use that do not vary over time. Other approaches can be used to assess short-term exposures but suffer from less spatial resolution. This specific air pollution exposure indicator has been associated with bronchiolitis and asthma incidence in other studies in Vancouver (Clark et al. 2007; Demers et al. 2006), and the same exposure assessment approach has been used in other cities to predict health outcomes such as asthma incidence, allergies, and wheezing (Brauer et al. 2002, 2007; Ryan et al. 2007).

### Psychosocial measure

We assessed chronic stress in children using the UCLA Life Stress Interview (Hammen 1991). This interview was conducted at baseline and assessed chronic stress over the preceding 6 months in domains such as family relationships, friendships, and school. This is a semistructured interview in which a trained interviewer asks a series of open-ended questions in each life domain and uses the information gathered to rate the level of chronic, ongoing stress. Interviewers rate the extent of chronic stress on a 1–5 scale, using 0.5 increments, with higher numbers reflecting more severe, persistent difficulties. This interview has been used successfully in children as young as 8 years of age and has demonstrated reliability and validity (Adrian and Hammen 1993; Rudolph and Hammen 1999). In the present study we focused on chronic family stress because family stress, of all the life stress domains, is the one most strongly related to asthma outcomes (Chen et al. 2006; Miller and Chen 2006).

### Immune measures

At the baseline visit, peripheral blood was drawn from children into BD vacutainer cell preparation tubes containing sodium heparin, and 3 × 10^6^ peripheral blood mononuclear cells (PBMCs) were isolated through density-gradient centrifugation. PBMCs were resuspended in culture medium consisting of RPMI plus 10% fetal calf serum and incubated with phorbol myristate acetate (25 ng/mL) and ionomycin (1 μg/mL) for 48 hr at 37°C in 5% carbon dioxide . Supernatants were frozen until the end of the study and were then assayed to determine levels of interleukin (IL)-4, IL-5, and IL-13 using enzyme-linked immunosorbent assays (assays from R&D Systems, Minneapolis, MN, USA). Intraassay coefficients of variation ranged from 3.68 to 4.76%.

We performed a complete blood count with five-part differential (Bayer ADVIA 70 hematology system; Holiston, MA, USA) to obtain eosinophil counts. Total serum immunoglobulin E (IgE) was measured using an automated fluorescence immunoassay (Pharmacia CAP system, Portage, MI, USA), and was log transformed because of its non-normal distribution.

### Clinical measures

To obtain multiple perspectives on asthma symptoms, both parents and children were probed about symptoms. Parents were interviewed during the laboratory visit about their child’s symptoms over the previous 2 weeks as part of a health interview that gathered information about the child’s asthma (e.g., prescribed medications). The frequency of daytime symptoms, nighttime symptoms, and exertional symptoms were probed according to the National Asthma Education and Prevention Program Expert Panel Report 2 (NAEPP/ EPR2) guidelines (NAEPP 1997). We assessed daytime symptoms as the number of days over the previous 2 weeks children had cough, wheeze, shortness of breath, or chest tightness. We assessed nighttime symptoms as the number of days children awakened from sleep because of coughing, wheezing, shortness of breath, or chest tightness. And we assessed exertional symptoms as the number of days children had cough, wheeze, shortness of breath, or chest tightness while exercising or playing.

Children were asked to rate their symptoms on a daily basis for 2 weeks after their laboratory visit. Children were asked to keep a diary of their symptoms, and every morning and evening they rated the extent of coughing, wheezing, chest tightness, and shortness of breath, each on a 0 (none) to 4 (really bad) scale. Scores for each question were summed for each day, then averaged across the 14-day diary period.

Over the same period, children also monitored peak expiratory flow rate (PEFR) at home using an electronic monitor (Quadromed, Hoechberg, Germany). Three peak flow readings were taken on awakening and before bedtime each day for 2 weeks, and the highest value at each time point was retained. Daily PEFR% was calculated as a percent of each youth’s laboratory best, and readings across the 2 weeks were averaged.

All clinical measures were repeated 6 months after the baseline visit.

### Potential confounders

Variables that could provide alternative explanations for the above relationships were included as covariates in statistical analyses. This included asthma severity, determined from the NAEPP/EPR2 guidelines based on the higher of symptom frequency and medication use, paralleling the approach of previous researchers (Bacharier et al. 2004). Families also brought children’s asthma medications to the research center, and inhaled corticosteroid use was coded (number of days taken during the preceding 2 weeks), as was beta agonist use (number of days taken during the preceding 2 weeks).

In addition, we assessed whether demographic characteristics or study visit variables, such as child age, sex, ethnicity, or time of year of study visit were associated with study variables. Demographic or study variables that were significantly associated with any immune or clinical measures were included as covariates in analyses with that outcome.

### Statistical analyses

We conducted statistical analyses to test the hypothesis that psychologic stress would interact with air pollution exposure to predict both biologic and clinical asthma outcomes. Biologic variables were examined cross-sectionally and clinical outcomes longitudinally, using a series of hierarchical multiple regression analyses. Biologic variables were predicted from variables entered in three steps: *a*) potential confounders including medical variables (asthma severity classification, use of inhaled corticosteroids, use of beta agonists) and any demographic variable associated with study outcomes; *b*) main effects of chronic stress and air pollution; and *c*) the interaction term for chronic stress by air pollution. These analyses were conducted according to the recommendations by Aiken and West (1991). Because both chronic stress and pollution exposure are continuous variables, these involve statistical procedures to examine the significance of an interaction between two continuous variables. These analyses comprised the primary test of study hypotheses. The nature of an interaction between two continuous variables, however, can be difficult to visualize. To aid in interpretation, we provided two additional pieces of information. Significant interactions were plotted by graphing the relationship between stress and asthma outcomes at low (−1 SD) and high (+1 SD) levels of air pollution. This creates an artificial distinction within one of the continuous variables, but allows one to more easily see how the relationship between stress and asthma varies at different levels of pollution exposure. We also conducted secondary analyses to test whether the regression coefficients at these specific values of air pollution were significant. Again, this creates an artificial distinction, but allows the reader to gain some sense of where the differences are most pronounced. These procedures follow the statistical recommendations of Aiken and West (1991). However, because these analyses do not fully represent the nature of the study variables (as continuous), they are considered secondary, with the test of the interaction between the two continuous variables as the primary analyses.

Clinical variables included two time points spaced 6 months apart. In these analyses, the difference score (time 2 − time 1) was predicted from the same set of variables described above, except that time 1 values were included as a control variable in step 1.

## Results

Table 1 presents descriptive information about the sample. There were a few associations of demographic and study variables with biologic or clinical outcomes. Child age was inversely correlated with eosinophil count (*r* = −0.23, *p* = 0.04), positively correlated with daily diary symptoms (*r* = 0.25, *p* = 0.04), and positively correlated with PEFR% (*r* = 0.24, *p* = 0.04). Parents reported girls to have more symptoms than boys (*t* = 3.36, *p* = 0.001). Children belonging to minority groups had higher production of IL-4 compared with white children (*t* = 3.40, *p* = 0.001). Time of year was correlated with IL-13 production (*r* = −0.22, *p* = 0.05), such that higher levels were found for those children seen during the winter months. Variables associated with outcomes were included as covariates in the relevant equations below.

### Cross-sectional associations with biologic markers

Our first set of analyses tested whether stress and air pollution were associated with biologic markers implicated in asthma. Regression coefficients are reported below, with interactions graphed to illustrate the direction of effects.

**Figure 1:**
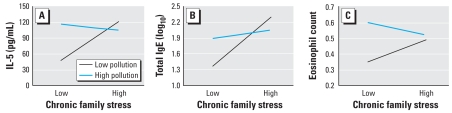
(*A*) Interaction between chronic stress and air pollution predicting IL-5 production. The graph displays the estimated regression line for the relationship between chronic stress and IL-5 production at low (−1 SD) and high (+1 SD) levels of air pollution. (*B*) Interaction between chronic stress and air pollution for total IgE. (*C*) Interaction between chronic stress and air pollution for eosinophil counts.

With respect to IL-5 production, there was no main effect of stress (β = 0.16, *p* = 0.22) or air pollution (β = 0.15, *p* = 0.30), but there was a significant stress by air pollution interaction (interaction term β = −0.31, *p* = 0.02). The negative coefficient indicates that as pollution decreases, higher levels of chronic family stress become associated with greater production of IL-5. This is illustrated in Figure 1A by plotting the relationship between chronic stress and IL-5 production at low and high levels of air pollution. The relationship between stress and IL-5 production was positive and statistically significant at 1 SD below the mean of air pollution (β = 0.39, *p* = 0.03), whereas the relationship between stress and IL-5 production at 1 SD above the mean of air pollution was not significant (β = −0.06, *p* = 0.71), suggesting that the relationship between stress and IL-5 production is stronger in lower-pollution areas.

No significant associations emerged for IL-4 or IL-13 (*p* > 0.2).

With respect to total IgE, there was a significant main effect of stress such that children with higher chronic stress had higher IgE levels (β = 0.32, *p* = 0.01), but no main effect of air pollution (β = 0.08, *p* = 0.52). In addition, there was a significant stress by air pollution interaction (interaction term β = −0.29, *p* = 0.02). The negative coefficient indicates that as pollution decreases, higher levels of chronic family stress become associated with greater IgE levels. This is illustrated in Figure 1B by plotting the relationship between chronic stress and IgE at +1 SD and −1 SD of air pollution. The relationship between stress and IgE was positive and statistically significant at 1 SD below the mean of air pollution (β = 0.54, *p* = 0.002), whereas the relationship between stress and IgE at 1 SD above the mean of air pollution was not significant (β = 0.11, *p* = 0.46), suggesting that the relationship between stress and IgE is stronger in lower pollution areas.

With respect to eosinophil counts, there was no main effect of stress (β = 0.05, *p* = 0.67), and a weak effect of air pollution (β = 0.21, *p* = 0.10). There was a significant stress by air pollution interaction (interaction term β = −0.24, *p* = 0.04). The negative coefficient indicates that as pollution decreases, higher levels of chronic family stress become associated with greater eosinophil counts. This is illustrated in Figure 1C by plotting the relationship between chronic stress and eosinophil counts at +1 SD and −1 SD of air pollution. The relationship between stress and eosinophil counts was positive at 1 SD below the mean of air pollution (β = 0.23, *p* = 0.15), whereas the relationship between stress and eosinophil counts at 1 SD above the mean of air pollution was negative (β = −0.13, *p* = 0.35).

### Longitudinal associations with clinical outcomes

Table 2 presents descriptive information on clinical variables at baseline and follow-up on the sample. This information is separated into a low- and a high-pollution group by median split so readers can see how clinical variables vary by pollution. Average (± SD) pollution exposure for those below the median was 14.1 ± 1.6 ppb. Average exposure for those above the median was 18.9 ± 3.5 ppb. Low-and high-pollution groups were similar on demographic variables such as age (average age for those below the median on pollution exposure = 12.2 years; average age for those above the median = 13.5 years), ethnicity (66% white among those below the median on pollution exposure; 61% white among those above the median), and parent education (average years of education for those below the median on pollution exposure = 15.4; average years of education for those above the median = 15.6). The table reveals that children in high pollution areas had higher child-reported daily symptoms and parent-reported symptoms at both time points than those in low-pollution areas. In the analyses below, we focused on change over time in clinical variables to assess whether the cross-sectional associations with biologic markers have implications clinically for asthma over time. To do this, we tested whether the interaction between stress and air pollution predicted changes in clinical variables over a 6-month period, controlling for baseline levels.

**Figure 2:**
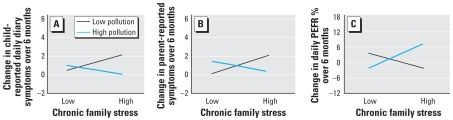
(*A*) Interaction between chronic stress and air pollution predicting change in child-reported daily diaries of asthma symptoms over a 6-month period. The graph displays the estimated regression line for the relationship between chronic stress and changes in asthma symptoms over time at low (−1 SD) and high (+1 SD) levels of air pollution. (*B*) Interaction between chronic stress and air pollution for changes in parent-reported symptoms over a 6-month period. (*C*) Interaction between chronic stress and air pollution for changes in daily PEFR percent over a 6-month period.

With respect to the daily diaries that children kept of symptoms, there was no main effect of stress (β = 0.06, *p* = 0.63) or air pollution (β = −0.12, *p* = 0.38), but there was a significant stress by air pollution interaction (interaction term β = −0.28, *p* = 0.02). The negative coefficient indicates that as pollution decreases, higher levels of chronic family stress become associated with increasing symptoms over time. This is illustrated in Figure 2A by plotting the relationship between chronic stress and change in symptoms at +1 SD and −1 SD of air pollution. The relationship between stress and symptom change was positive at 1 SD below the mean of air pollution (β = 0.25, *p* = 0.098), whereas the relationship between stress and symptom change at 1 SD above the mean of air pollution was negative (β = −0.13, *p* = 0.35).

With respect to parent report of child symptoms, there was no main effect of stress (β = 0.08, *p* = 0.60) or air pollution (β = −0.03, *p* = 0.85), but there was a marginal stress by air pollution interaction (β = −0.25, *p* = 0.07). The negative coefficient indicates that as pollution decreases, higher levels of chronic family stress become associated with increasing parent-reported symptoms over time. This is illustrated in Figure 2B by plotting the relationship between chronic stress and change in symptoms at +1 SD and −1 SD of air pollution. The relationship between stress and symptom change was positive at 1 SD below the mean of air pollution (β = 0.36, *p* = 0.08), whereas the relationship between stress and symptom change at 1 SD above the mean of air pollution was negative (β = −0.21, *p* = 0.36).

With respect to daily PEFR measures, there was no main effect of stress (β = 0.05, *p* = 0.68) or air pollution (β = 0.06, *p* = 0.70), but there was a significant stress by air pollution interaction (β = 0.30, *p* = 0.03). The positive coefficient indicates that as pollution decreases, higher levels of chronic family stress also become associated with decreasing PEFR over time. This is illustrated in Figure 2C by plotting the relationship between chronic stress and change in PEFR at +1 SD and −1 SD of air pollution. The relationship between stress and PEFR change was negative at 1 SD below the mean of air pollution (β = −0.14, *p* = 0.40), whereas the relationship between stress and PEFR change at 1 SD above the mean of air pollution was positive (β = 0.24, *p* = 0.11).

Although the direction of change differed by pollution levels, this does not mean that symptoms are actually higher in lower-pollution areas. As shown in Table 2, children above the median in pollution exposure had greater symptoms by daily diary report and parent report than did children below the median in pollution exposure, and had comparable PEFRs. Hence, children in higher-pollution areas have greater symptoms, but these symptoms do not appear to worsen over time the way they do for children in lower-pollution areas with chronic stress.

## Discussion

This article is the first that we are aware of to document that physical environment (chronic traffic-related air pollution) and social environment (chronic stress) interact to predict both biologic and longitudinal clinical outcomes in children with asthma. The findings from this study demonstrated that the interactive effects between air pollution and stress are stronger than either factor alone, suggesting that the physical and social environments are in fact intertwined and critical to understand in concert, rather than independently.

The nature of this interaction was such that the detrimental effects of chronic psychosocial stress were more evident among children living in lower-pollution areas. That is, as pollution levels declined, higher levels of stress were associated with heightened inflammatory profiles cross-sectionally and worsening clinical profiles over a 6-month period. In contrast, chronic stress had modest effects on biologic and clinical measures as pollution exposure increased, and any suggestions of effects were in an opposite direction.

The direction of the interaction effects in this study was different from that found in the small number of previous studies on this topic. For example, one recent study found that traffic-related air pollution (NO_2_) interacted with exposure to violence to predict the diagnosis of asthma in a birth cohort of children, such that children with both high pollution and violence exposures were at greatest risk of having asthma (Clougherty et al. 2007). Another study found that high levels of traffic-related air pollution (e.g., NO_2_) combined with low socioeconomic status predicted the greatest risk of asthma hospitalizations in children (Lin et al. 2004). These previous studies fit a “double jeopardy” hypothesis, suggesting that the combination of physical and social exposures synergistically affect asthma outcomes.

In contrast, our data suggest that chronic stress may have the ability to accentuate the effects of environmental pollutants when chronic exposure levels are more modest. Previous research has suggested that social factors do require the presence of some dose of physical exposure to have effects on biologic processes (Chen and Miller 2007). The findings from the present study fit the notion of a threshold model—that is, that there is a threshold at which chronic physical exposures begin to have effects on health outcomes, and that one role of chronic stress may be to lower the threshold at which physical exposures affect biologic and clinical outcomes. One reason why this may occur is that when chronic exposure to traffic-related air pollutants is more modest, there may be greater room for social factors to increase or decrease vulnerability biologically. This is consistent with the notion that stress may be able to shift physiologic response systems in a direction such that adverse outcomes occur in response to lower doses of physical exposures (Morello-Frosch et al. 2006; Paarlberg et al. 1995). Consistent with this notion, chronic stress under certain conditions has been found to heighten biologic responses to negative social exposures (Gump and Matthews 1999); we speculate that similar processes may occur with responses to physical exposures. This type of response pattern is thought to occur because prolonged stress can sensitize and prevent adaptation of biologic systems (McEwen 1998), potentially leading to lower doses of physical environment pollutants having detrimental effects on biologic and clinical asthma measures.

Reasons for the differences between our study and the two studies cited above are unclear, but we speculate that they may be attributable to distinctions between the diagnosis versus progression of a disease, or to different conceptualizations of social exposures. Our study focused on children with preexisting asthma and predicted biologic outcomes as well as changes in clinical outcomes over time; in contrast, the study by Clougherty et al. (2007) predicted the risk of being diagnosed with asthma. The way in which physical and social exposures affect the onset versus progression of disease could be different, resulting in the distinct patterns found in the two studies. In addition, different types of social exposures may have different effects on asthma. Although they all are forms of stress, exposure to violence, chronic family stress, and low socioeconomic status each represents different types of life stressors, and it is possible that air pollution interacts differently with different types of stressors. In the present study, we focused on family stress because this type of stress has the most robust associations with asthma outcomes (Chen et al. 2006; Miller and Chen 2006); nonetheless, it is possible that other unmeasured stressors, such as living in impoverished neighborhoods, also contribute to asthma biologic and clinical outcomes and overlap with air pollution indicators. Future research should test these possibilities further.

The effects that we found cross-sectionally of interactions between chronic traffic-related air pollution and chronic stress on IL-5 production, total IgE levels, and eosinophil counts represent biologic pathways that have implications for clinical asthma outcomes. Immune pathways in asthma include the secretion of cytokines that activate B cells to produce IgE. IgE in turn initiates an inflammatory cascade leading to airway constriction and mucus production. A second pathway involves the recruitment of eosinophils to the airways, which also promotes airway inflammation and obstruction. Secretion of the cytokine IL-5 is known to increase eosinophil production. Thus heightened production of IL-5 along with elevated IgE and eosinophil counts suggests a biologic profile that is potentially detrimental for children with asthma in terms of vulnerability to symptoms.

We considered this possibility by testing whether chronic traffic-related air pollution by chronic stress interactions could also predict changes in clinical outcomes over a 6-month period. Consistent with the implications of the cross-sectional biologic data, we found that in lower-pollution areas, higher levels of chronic stress at baseline predicted increases in asthma symptoms and decreases in daily PEFR over time. Hence chronic stress appears to exacerbate the effects of more modest exposures to chronic air pollutants on longer-term clinical asthma outcomes, in addition to biologic markers.

Interestingly, as pollution levels increased, an opposite pattern emerged whereby higher levels of chronic stress were associated with declines in asthma symptoms and increases in PEFR over time. This was an unexpected trend, and it is unclear what the implications are. However, because longitudinal analyses focus on change over time, this does not mean that children in higher-pollution areas have absolute levels of asthma morbidity that are low. Rather, children in higher-pollution areas have more symptoms than children in lower-pollution areas at both time points, but their clinical profiles (as assessed by PEFR and symptom reporting) do not appear to worsen over time. In contrast, children in lower-pollution areas show stronger relationships of chronic stress with worsening clinical profiles over time.

Strengths of the present study include the collection of asthma-relevant biomarkers; the tracking of longitudinal clinical outcomes; the use of a land-use regression model to assess individual exposures to air pollution; and the use of an in-depth interview for measuring chronic stress. In addition, the design of the study meant that directionality could be more clearly inferred. For example, although it would be reasonable to hypothesize that more severe asthma increases family stress levels, the fact that stress was assessed before clinical measures for longitudinal analyses meant that worsening asthma was not driving stress experiences.

Limitations to the present study include the small sample size. Both the comprehensive stress interview and the collection and processing of biologic samples limited the size of the present sample, and this raises the possibility that findings may have been attributed to chance and hence need to be replicated. However, our sample size is comparable to those of numerous other studies of acute stress and asthma cytokine production (Kang and Fox 2001; Kang et al. 1997; Marshall et al. 1998). A second limitation is the varying time frame for measures in this study. Time frames were set based on optimal periods for gathering information for different study constructs. For example, symptom reports are best assessed over shorter intervals (weeks), given the difficulties in accurately recalling symptoms over longer time periods (NAEPP 1997). Chronic stress is best assessed over a period of months to accurately capture persistent stressful influences in different life domains (Hammen 1991). Finally, pollution estimates using land use regression models are best suited for long-term exposures, given that the model is based on spatial differences in land use that do not vary over time. Because pollution data were available for a 6-year period (1998–2003), but this period did not overlap with the time frames of the other study constructs, we used the entire period as a more reliable indicator of long-term exposures. One limitation of this approach is that if families moved, the estimated exposures would be misclassified; however, this increase in measurement error would be expected to decrease the likelihood of observing associations. Nonetheless, future studies that *a*) are able to more precisely coordinate the periods of assessment for air pollution, stress, and clinical outcomes, and *b*) could repeatedly assess families to track changes in pollution exposures based on moves, as well as changes in chronic stress experiences over time, would be useful for more clearly delineating the time frame of effects of physical and social exposures. A third limitation is the lack of health records to ascertain objective asthma-relevant outcomes such as hospitalizations and physician visits. Future studies that have access to such databases would allow researchers to explore additional clinical indicators that may be influenced by both the physical and social environments. Finally, we used NO_2_ as an indicator of traffic-related air pollution, and effects may be attributable specifically to NO_2_. Further, although we assessed pollution exposure with a high-resolution spatial model estimating air pollution concentrations at the individual subjects’ home address, we did not consider short-term temporal variability in exposures during the study period, nor did we consider other environments (e.g., schools) where participants spend a good deal of time. Because it was not feasible to measure the actual level of pollution exposure that each child experienced throughout the day, this raises the possibility that some of the patterns could have been affected by unmeasured exposures, particularly in a small sample such as this one.

In summary, in this study we found that the physical and social environment interact to affect asthma outcomes in children. As pollution levels declined, higher levels of chronic family stress were associated with heightened inflammatory profiles cross-sectionally and with increases in asthma symptoms and decreases in peak expiratory flow over a 6-month period. Conversely, as pollution levels increased, chronic stress either had no effect on outcomes (inflammatory measures) or in some cases showed an opposite effect (clinical measures). These findings suggest that vulnerability factors such as psychosocial stress most clearly modify the effects of traffic-related air pollution when exposure is present but not high. This highlights the need for families who have a child with asthma to be increasingly aware of how the physical and social environments are intertwined, and how vulnerability to asthma exacerbations may persist in children who are experiencing higher levels of chronic stress, even in areas with lower levels of pollution.

## Figures and Tables

**Table 1:** Descriptive information on study participants.

Characteristic	Value
Age	12.82 ± 2.75
Sex (%)	
Male	68
Female	32
Ethnicity (%)	
White	63
Asian	26
Other	11
Severity (%)	
Mild intermittent	16
Mild persistent	38
Moderate persistent	32
Severe persistent	14
Inhaled corticosteroids^a^	4.35 ± 5.91
Beta agonists^a^	3.93 ± 5.55
Chronic stress^b^	2.14 ± 0.74
NO_2_ (ppb)	16.5 ± 3.7
IL-4 (pg/mL)	12.47 ± 10.85
IL-5 (pg/mL)	110.46 ± 91.00
IL-13 (pg/mL)	323.42 ± 237.22
IgE (log-transformed kU/L)	2.18 ± 0.80
Eosinophil count (× 10^9^cells/L)	0.36 ± 0.28
Parent-reported symptoms^c^	
Baseline	1.96 ± 2.79
6-month follow-up	1.23 ± 2.08
Child-reported daily diary symptoms^d^	
Baseline	3.42 ± 3.85
6-month follow-up	2.61 ± 3.23
Daily peak expiratory flow rate (%)	
Baseline	99.83 ± 15.62
6-month follow-up	95.08 ± 16.64

Values are mean ± SD except where indicated.

a Medication values are for number of days taken in the preceding 2 weeks.

b Chronic stress is on a 1–5 scale.

c Parent-reported symptoms = average number of days of symptoms in preceding 2 weeks reported during the laboratory visit.

d Child-reported daily diary symptoms = average daily symptom score from the 2-week home monitoring after the laboratory visit.

**Table 2:** Descriptive information on clinical measures by pollution group (mean ± SD).

	Low pollution^a^	High pollution^b^
Child daily diary symptoms, baseline	2.79 ± 3.42	4.08 ± 4.21
Child daily diary symptoms, follow-up	2.09 ± 2.35	3.16 ± 3.90
Parent-reported symptoms, baseline	3.47 ± 4.44	8.47 ± 10.67
Parent-reported symptoms, follow-up	2.91 ± 6.22	4.50 ± 6.26
PEFR%, baseline	100.02 ± 16.07	99.62 ± 15.35
PEFR%, follow-up	94.15 ± 12.93	96.07 ± 20.04

a Those below the median on NO_2_ scores.

b Those above the median on NO_2_ scores.
